# The course of fatigue during the development of rheumatoid arthritis and its relation with inflammation: a longitudinal study

**DOI:** 10.1016/j.jbspin.2022.105432

**Published:** 2022-06-28

**Authors:** Sarah J.H. Khidir, Fenne Wouters, Annette H.M. van der Helm-van Mil, Elise van Mulligen

**Affiliations:** a Department of Rheumatology, Leiden University Medical Center, 2300 RC Leiden, The Netherlands; b Department of Rheumatology, Erasmus Medical Center, Rotterdam, The Netherlands

**Keywords:** Fatigue, Rheumatoid arthritis, Clinically suspect arthralgia, Time course, Patient reported outcomes

## Abstract

**Objective:**

Fatigue is a prominent and disabling symptom in patients with rheumatoid arthritis (RA), that is only partially explained by inflammation and responds poorly to DMARD-therapy. We hypothesized that inflammation explains fatigue to a larger extent in the phase of clinically suspect arthralgia (CSA), when persistent clinical arthritis is still absent and fatigue has not yet become chronic. We therefore studied the course of fatigue in CSA during progression to RA and the association with inflammation at CSA-onset and at RA-diagnosis.

**Methods:**

600 consecutive CSA-patients were followed for RA-development. Additionally, 710 early RA-patients were studied at diagnosis. Fatigue was assessed every study visit and expressed on a 0-100 scale. Inflammation was measured with the DAS44-CRP, with and without including subclinical inflammation. The course of fatigue over time was studied with linear mixed models. Associations between fatigue and inflammation were studied with linear regression. Analyses were stratified by ACPA-status.

**Results:**

In 88 CSA-patients who developed RA, pre-arthritis fatigue-levels increased gradually with 7 points/year, towards 48 (95%CI=41-55) at RA-development (*P*=ns). Fatigue decreased in CSA-patients who did not develop RA (4 points/year, *P*<0.001). At CSA-onset, inflammation was associated with fatigue (β=18, meaning 18 points more fatigue per point increase DAS-score, *P*<0.01). This association was stronger than at RA-diagnosis (β=5, *P*<0.001). Fatigue-levels were lower in ACPA-positive pre-RA, but its association with inflammation was stronger compared to ACPA-negative pre-RA.

**Conclusion:**

Fatigue increased gradually during progression from arthralgia to clinical arthritis, and fatigue was better explained by inflammation in CSA than in RA. This implies a ‘phase-dependent relation’ between inflammation and fatigue.

## 1 Introduction

Fatigue is a major hurdle for patients with rheumatoid arthritis (RA). Whereas many outcomes of RA have improved over the past two decades, the severity of fatigue in RA has not diminished [1]. This is worrisome since RA-patients indicate that fatigue is one of the most important disease burdens [2,3].

Treat-to-target strategies aim at suppressing inflammation. The absence of effects of such strategies on fatigue may be explained by the finding that inflammation and fatigue in RA are poorly and inconsistently correlated [4]. Previous studies in RA showed that higher levels of inflammation, measured with the Disease Activity Score (DAS), associated with higher levels of fatigue, and that a decline in DAS was followed by a decrease in fatigue [1,5]. However, effect sizes were small and fatigue could only be partially explained by inflammation. This implies that the etiology of fatigue in RA is multidimensional and includes not only inflammation, but also cognitive and behavioral factors (e.g. thoughts, feelings, and behaviors), and personal factors (e.g. work/caring responsibilities, social support and environment) [6]. These non-inflammatory factors are not targeted with treat-to-target treatment.

After RA-diagnosis, when symptoms have been enduring, non-inflammatory factors and fatigue may have become captured in a circular process [4]. This could contribute to the persistency of fatigue. In addition, it remains possible that immunological pathways negatively affect long term changes in brain functioning in areas that are involved in fatigue [7]. This could explain the limited role of inflammation on fatigue in the phase of RA, when disease processes are presumably matured. However, it is known that immune and inflammatory processes in RA start gradually and already before clinical arthritis becomes apparent, and thus before RA can be diagnosed [8]. We hypothesized that inflammation explains fatigue to a larger extent in the pre-arthritis phase of RA, when RA is still developing and fatigue has not yet become chronic. This hypothesis prompted us to perform the current study. In patients with clinically suspected arthralgia (CSA), when clinical arthritis is still absent, we determined the course of fatigue. Furthermore, the associations between fatigue and inflammation were studied at CSA-onset and at RA-diagnosis.

## 2 Methods

### 2.1 Patients

We longitudinally studied CSA-patients who were consecutively included in the Leiden CSA-cohort and had at least one year of follow-up. The CSA-cohort is an inception cohort including patients with arthralgia of the small joints for less than one-year that is considered suspicious for progression to RA (Data S1) (Supplementary data) [9]. Patients were not included if the rheumatologist considered another explanation for the arthralgia (e.g. osteoarthritis or fibromyalgia) more likely than imminent RA. Patients were followed for two years for development of clinical arthritis, confirmed with joint swelling at physical examination by the rheumatologist. Follow-up visits were performed at 4, 12 and 24 months and more regularly in case of any increased musculoskeletal symptoms between follow-up visits. During follow-up, CSA-patients were not treated with DMARDs (including corticosteroids).

For the current research, CSA-patients who progressed to RA were analyzed separately from those who did not progress to RA. RA was defined as clinical arthritis plus fulfillment of the 1987- and/or 2010-criteria for RA, or clinical arthritis with a clinical diagnosis and start of DMARD-treatment [10]. The latter group was included in this definition because CSA-patients had easy access to rheumatological care, and rapid DMARD-start after the occurrence of clinical arthritis may have prohibited progression towards fulfillment of the criteria.

Patients at diagnosis of RA were obtained from the Leiden early arthritis clinic (EAC) cohort (Data S2). The EAC-cohort includes consecutive patients with clinical arthritis at physical examination by the rheumatologist and a symptom duration of <2 years. Patients included between August 2010 and March 2020, and fulfilling the 1987- and/or 2010-criteria for RA within one year, were evaluated in the present study. All patients in the CSA- and EAC-cohort provided written informed consent.

### 2.2 Fatigue

Experienced fatigue was assessed uniformly by a trained research nurse at every visit and was expressed on a scale from 0 (no fatigue) to 100 (extreme fatigue), with the question “How tired were you the last day?”. The fatigue-scale has previously been validated for use in RA, and has a minimal clinically important difference (MCID) of 10 [11,12].

### 2.3 Inflammation

Inflammation was measured in two ways. In the primary analysis the three variables (3 v) variant of the DAS44-CRP was used. The DAS44-CRP(3 v) is composed of the swollen joint count (SJC), CRP-level and tender joint count (TJC). The patient’s global assessment (PtGA) is only included in the DAS(4 v), but this DAS-score was considered inappropriate because fatigue and the PtGA are strongly correlated [13]. In the absence of a validated score to use in CSA, when clinical arthritis is per definition absent, we applied the DAS44-CRP with SJC=0.

Secondly, we repeated the analyses after replacing the SJC in the original DAS by the number of hand/feet joints with magnetic resonance imaging (MRI-)detected subclinical inflammation (synovitis, tenosynovitis or osteitis), which was defined as inflammation that was present in <5% of age matched healthy controls at the same location (Data S3). In this study this was called ‘DAS-adjusted’. Sub-clinical inflammation was assessed with contrast-enhanced 1.5T MRI of the wrist, metacarpophalangeal (MCP(2-5)) and metatarsophalangeal (MTP(1-5))-joints at baseline (Data S4). All MRIs were scored semi-quantitatively for inflammation in line with the validated Rheumatoid Arthritis Magnetic Resonance Imaging Score (RAMRIS) and the method of Haavardsholm (Data S3). Inter- and intra-reader intraclass correlation coefficients were ≥0.90.

### 2.4 Statistical analyses

CSA-patients who developed RA during follow-up are in retrospect truly ‘pre-RA’ when presenting with arthralgia. The course of fatigue in the CSA-patients with RA-development was studied with linear mixed models with time before clinical arthritis-development as independent variable. In this analysis, date of clinical arthritis was set to timepoint zero for every patient individually and all fatigue-levels prior to their clinical arthritis were studied, while accounting for repeated data within individuals. No other variables than time before arthritis and fatigue levels during follow-up were included in this linear mixed model analysis. A separate LMM-analysis was performed in the group of CSA-patients not developing RA, but with time after baseline-visit as independent variable.

Association between fatigue and inflammation at baseline was studied by linear regression analysis with fatigue as outcome and DAS44-CRP as independent factor, corrected for sex. This association was assessed in separate models for CSA-patients developing RA at CSA-onset and for RA-patients at RA-diagnosis. Analyses were stratified by anti-citrullinated protein antibodies (ACPA-)status. A summary scheme of statistical analyses is provided in Figure S1. All analyses were performed using SPSS v25 and two-sided p-values of <0.05 were considered statistically significant.

## 3 Results

### 3.1 Patient characteristics

**Fig. 1:**
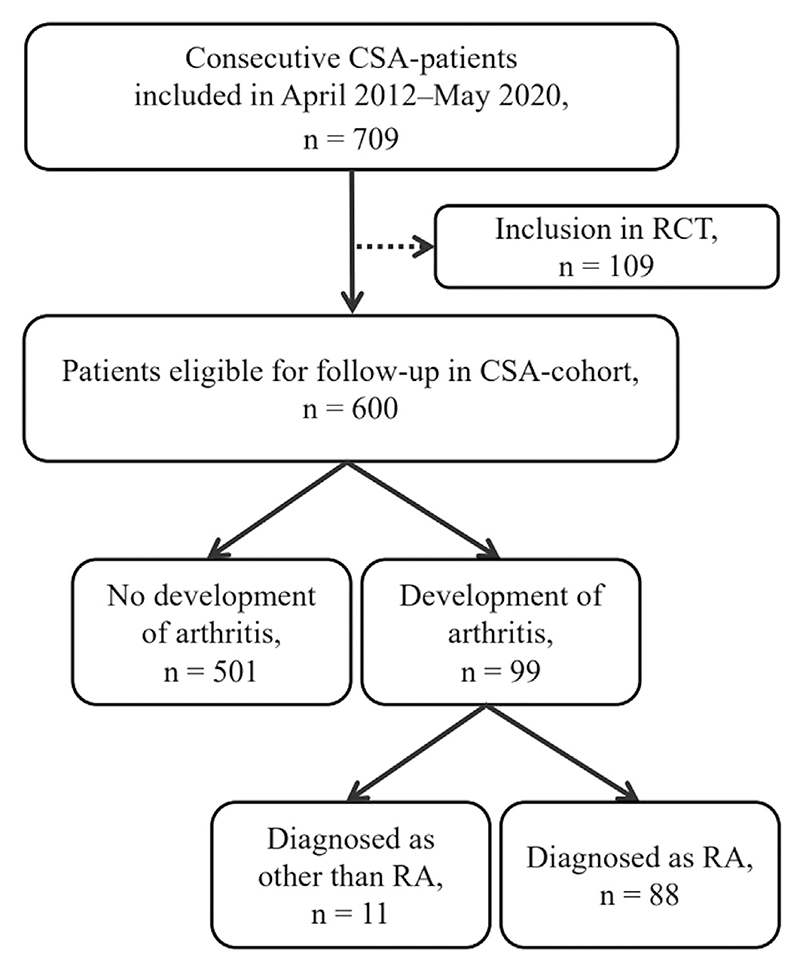
Flow diagram of study inclusion of CSA-patients. 109 patients were excluded from analyses because of participation in the Treat-Earlier Trial studying whether methotrexate can prevent progression to RA. These patients were excluded from the current study due to 50% chance of methotrexate use [21]. 11 patients developed arthritis during follow-up, but were diagnosed as other than RA: undifferentiated arthritis (*n*=8), psoriatic arthritis (*n*=2) and osteoarthritis (*n*=1). These eleven patients were not included in CSA-analyses. CSA: clinically suspect arthralgia; RA: rheumatoid arthritis; RCT: randomized controlled trial.

In total, 600 CSA-patients were included and were followed for a median follow-up of 25 months (IQR 15-26), during which 88 developed RA (Fig. 1). Baseline characteristics of included patients are shown in Table 1. Out of 88 CSA-patients developing RA, 74% was female and mean age was 48 years. Out of 710 included early-RA patients, 63% was female and mean age was 59 years.

### 3.2 Change in fatigue over time in CSA

**Fig. 2:**
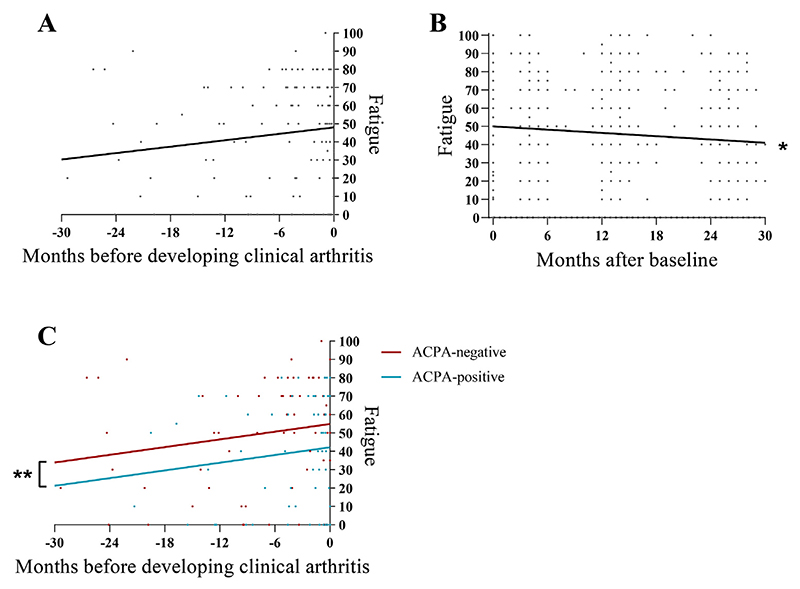
Course of fatigue in CSA-patients developing RA (A), in CSA-patients not developing RA (B), and in CSA-patients developing RA stratified by ACPA-status (C). Lines depict the average course derived from linear mixed model analysis. Timepoint zero was defined as time of clinical arthritis in CSA-patients who developed RA, and as the first visit in CSA-patients who did not develop RA. Fig. 2B contains data of 475 patients at baseline (t=0), but most dots overlap. Fatigue increased in CSA-patients who developed RA with 7 points/year, p=ns. *Fatigue decreased in CSA-patients who did not develop RA with 4 points/year, *P*<0.001. **ACPA-negative CSA-patients were more fatigued than ACPA-positive, with a mean difference in fatigue of 13 points, *P*<0.05. ACPA: anti-citrullinated protein antibody; CSA: clinically suspect arthralgia; RA: rheumatoid arthritis.

In CSA-patients developing RA, pre-arthritis fatigue-levels increased gradually over time towards a mean of 48 (95%CI=41-55) at development of clinical arthritis. The mean increase in fatigue was 7 points per year (β=7, 95%CI= -2 to 16, p=ns; Fig. 2A). CSA-patients who did not develop RA, showed decreasing fatigue-levels of 4 points per year after baseline (β= -4, 95%CI= -6 to -3, *P*<0.001; Fig. 2B).

### 3.3 Association between fatigue and inflammation

In CSA-patients who progressed to RA, inflammation (DAS44-CRP) was significantly associated with severity of fatigue at the time of presentation with arthralgia; β=18, 95%CI=7-28, *P*<0.01; Table 2. A β of 18 indicates 18 points more fatigue per 1 point increase in DAS-score. Inflammation was also associated with fatigue at the time of RA-diagnosis, but the effect size was smaller: β=5, 95%CI=3-7, *P*<0.001, hence patients were 5 points more fatigued per 1 point increase in DAS-score. In other words, a point increase in DAS in CSA was associated with an increase in fatigue more than the MCID, whilst this was not the case at the time of RA-diagnosis. The extent to which fatigue could be explained (R^2^) by inflammation was 12.9% in CSA and 8.5% in early RA. Analyses were repeated with the ‘DAS-adjusted’ that included the number of subclinical inflamed joints. This showed comparable results to the association between DAS44-CRP and fatigue at CSA-onset and RA-diagnosis (Table 3). These results are obtained from linear regression analyses with correction for sex.

### 3.4 Stratification for ACPA

Stratified analyses by ACPA-status revealed that fatigue in ACPA-negative and ACPA-positive CSA-patients increased both in the same extent towards RA-development. However, ACPA-negative patients were more fatigued than ACPA-positive patients before and at development of RA (mean difference in fatigue of 13 points, 95%CI=1-24, *P*<0.05; Fig. 2C). Furthermore, fatigue was not significantly associated with inflammation in ACPA-negative CSA, whilst inflammation and fatigue were significantly associated in ACPA-positive CSA. Finally, the association between inflammation and fatigue in ACPA-positive CSA was stronger than in ACPA-positive RA (13=25 and 9, respectively; Table 2).

## 4 Discussion

Fatigue is a prominent and disabling symptom in RA-patients that is challenging to treat in clinical practice and is only partially explained by inflammation at the time of diagnosis and thereafter. Inflammatory processes however start long before clinical arthritis occurs. The course of fatigue in pre-RA is unknown, as well as the contribution of inflammation to fatigue in this disease phase. We hypothesized that inflammation plays a greater role on fatigue during pre-RA, when disease is not yet mature and fatigue has not yet become chronic. Therefore, we studied the course of fatigue during progression from CSA to RA, and the association of fatigue with inflammation at CSA-onset and at RA-diagnosis. We showed an increasing trend in fatigue over time during progression from CSA to RA. Furthermore, inflammation and fatigue were significantly associated in both disease stages, but the association was greater in the phase of arthralgia than at the time of clinical arthritis.

A change of 10 in fatigue is considered a clinically relevant difference [11]. An increase of 1 DAS-point was associated with a relevant increase in fatigue in CSA but not in RA. Although these results are estimates on group-level and are derived from conventionalized linear regression analyses, the results cannot be interpreted as an existing and linear association in all patients individually. Nevertheless, they seem to support our hypothesis that inflammation contributes more strongly to fatigue in the pre-arthritis phase of RA.

We found significantly higher levels of fatigue in ACPA-negative CSA-patients developing RA, in comparison to ACPA-positive patients. Our study validates previous findings on differences in fatigue-levels between ACPA-subgroups within RA, namely more severe fatigue in ACPA-negative RA, and extends this to the pre-RA phase [1,14]. Studying the association of fatigue with inflammation, surprisingly, we found no association in ACPA-negative CSA-patients that later-on developed RA, in contrast to ACPA-positive CSA-patients. These findings could support the hypothesis that different mechanisms underlie the development of ACPA-positive and ACPA-negative disease and disease related symptoms [15,16]. It is tempting to speculate that immunosuppressive or anti-inflammatory therapies will be more effective in reducing fatigue in ACPA-positive than ACPA-negative disease, but further research on this topic remains warranted.

This study is subject to some limitations, such as the absence of a validated inflammation score to be used in ‘pre-RA’. We used the DAS44-CRP with SJC=0 to quantify inflammation. Without SJC, the remaining components of the DAS are CRP and TJC. Even though TJC represents presumably pain, it also captures inflammation as the TJC has been accepted as a component of the DAS and has been shown to correlate with subclinical inflammation in CSA-patients [17]. Furthermore, the SJC receives scarce weight in the DAS-formula but one could still argue that subclinical inflammation is missed by setting the SJC on zero in CSA. We therefore performed the same analysis with the ‘DAS-adjusted’ including joints with MRI-detected inflammation, in both CSA and RA-patients. In the absence of a validated measure for inflammation in pre-RA, we believe that these options were best to summarize local and systemic inflammation into one score. Reassuringly, both measures of inflammation showed comparable findings on the association with fatigue in CSA and similar differences between the association strengths of inflammation and fatigue in the phase of CSA and RA.

A second limitation is that observed associations between inflammation and fatigue do not prove causation. Still, differences in association strengths as observed for the different disease phases (pre-RA, RA respectively) are suggestive of differences in factors contributing to fatigue. Moreover, we analyzed the course of fatigue in CSA-patients who did not develop RA during follow-up, from which a group has absence or resolution of subclinical inflammation [18]. The oppositional course of fatigue between CSA-patients with and without RA-development, reinforces the notion that inflammatory processes did contribute to fatigue in the phase of CSA.

A possible limitation in the used instrument for fatigue. Although the NRS fatigue is a quick and frequently used instrument for the assessment of fatigue, differences between assessment by research nurses and patients themselves have not been assessed in this study. Importantly however, fatigue was assessed in a similar manner in all patients and disease stages.

Finally, the follow-up duration of two years may be insufficient to identify all patients who converted to clinical arthritis. However, based on previous work, this risk is limited. In our study the majority of CSA-patients (86%) developed arthritis within one year and only 14% developed arthritis in the second year of follow-up. Previously, it has also been described that arthritis development after two years is infrequent [19].

There remains an unmet need for further research on the relation between fatigue and non-inflammatory factors in the phase of pre-RA. Although, we found effect sizes and explained variance of inflammation on fatigue to be larger in CSA than in RA, these were still moderate, emphasizing the multidimensional origin of fatigue. Of note, non-inflammatory factors that previously have been associated with fatigue such as anemia, age, comorbidities, sleep, exercise and mental health were not included in our study, but require acknowledgement in the overall comprehension of fatigue in (pre-)RA [1,6,20]. Non-inflammatory factors thus also contribute to fatigue in the pre-RA phase. The role of non-inflammatory factors and their potential bidirectional relation with inflammation and fatigue in pre-RA remains to be elucidated further [4].

In conclusion, to the best of our knowledge, this is the first study that analyzed fatigue over time and its relation with inflammation during RA-development. We showed an increasing trend of fatigue in CSA-patients towards RA-development. Furthermore, fatigue was more explained by inflammation at CSA-onset than at RA-diagnosis, and differences were observed between ACPA-positive and ACPA-negative disease. The stronger association with inflammation in the CSA-phase implies a ‘phase-dependent relation’ between inflammation and fatigue. These findings provide clues to unravel the complex pathophysiology of fatigue in (pre-)RA by using longitudinal data of patients from different phases of RA. Based on these findings, one could speculate that DMARD-treatment in the CSA-phase may diminish the burden of fatigue more effectively compared to the effect of DMARDs in classified RA, especially in ACPA-positive patients; though this remains a subject for future research.

## Supplementary Material

S1, S2, S3, S4

## Figures and Tables

**Table 1:** Patient characteristics. Data are *n* (%), mean ± SD or median (IQR). Please see the flowchart (Fig. 1); 11 patients with CSA developed clinical arthritis but not RA and were neither included in the group that developed RA, nor in the group that did not progress to RA.

	All CSA-patients, at CSA-onset (*n*=600)	CSA-patients developing RA, at CSA-onset (*n*=88)	CSA-patients not developing RA, at CSA-onset (*n*=501)	RA-patients, at diagnosis (*n*=710)
Female	469 (78)	65 (74)	398 (79)	448 (63)
Age in years	44 ± 13	48 ± 13	43 ± 12	59 ± 15
Symptom duration in days	137 (68-310)	153 (61-364)	134 (68-296)	93 (49-219)
Fatigue	51 ± 29	45 ± 29	52 ± 28	50 ± 30
Pain	47 ± 23	48 ± 27	47 ± 22	58 ± 25
TJC-53	5 (2-10)	5 (2-7)	5 (2-10)	8 (4-14)
CRP increased (≥5 mg/L)	130 (22)	32 (36)	96 (19)	468 (66)
DAS44-CRP	2.2 ± 0.7	2.1 ± 0.6	2.2 ± 0.7	3.5 ± 1.0
ACPA-positive	79 (13)	45 (51)	33 (7)	311 (44)
RF-positive	115 (19)	51 (58)	63 (13)	343 (48)
Number of small joints with imaging detected inflammation (0-10)	0 (0-1)	2 (1-3)	0 (0-1)	3 (1-5)

CSA: clinically suspect arthralgia; RA: rheumatoid arthritis; TJC: tender joint count; CRP: c-reactive protein; DAS: Disease Activity Score; ACPA: anti-citrullinated protein antibody; RF: rheumatoid factor; MRI: magnetic resonance imaging.

**Table 2:** Association between fatigue and inflammation (DAS44-CRP) at presentation with CSA in CSA-patients who later progressed to RA (A) and at RA-diagnosis (B). Results from linear regression analyses with fatigue (dependent factor) and disease activity defined by DAS44CRP (independent factor), corrected for sex. A β of 18 at CSA-onset indicates 18 points more fatigue per point increase in DAS-score. Likewise at RA diagnosis, patients have 5 point more fatigue per point increase in DAS. The minimal important difference in fatigue is known to be 10.

	CSA-onset	RA-diagnosis
All patients	β (95% CI) 18 (7–28)^a^	β (95% CI) 5 (3–7)^b^
ACPA-strati?ed:		
ACPA+	25 (7–42)^a^	9 (5 - 12)^b^
ACPA-	10 (-4, +25)	2 (-1, +5)

ACPA: anti-citrullinated protein antibody; CSA: clinically suspect arthralgia; RA: rheumatoid arthritis.

a *P*<0.01.

b *P*<0.001.

**Table 3:** Association between fatigue and inflammation (measured with the so-called ‘DAS-adjusted’) at presentation with CSA in CSA-patients who later on progressed to RA (A) and at RA-diagnosis (B). Results from linear regression analyses with fatigue (dependent factor) and disease activity defined by the so-called ‘DAS-adjusted’ (independent factor), corrected for sex. In all analyses using this ‘DAS-adjusted’, the swollen joint count was replaced by the number of joints with MRI-detected inflammation. A β of 13 indicates 13 points more fatigue per 1 point increase in DAS-adjusted score.

	CSA-onset	RA-diagnosis
All patients	β (95% CI) 13 (2 - 23)^a^	β (95% CI) 8 (4 - 11)^b^
ACPA-strati?ed:		
ACPA+	17 (0.5– 33)^a^	11 (6 - 17)^b^
ACPA-	8 (-7, +22)	4 (-0.5, +9)

ACPA: anti-citrullinated protein antibodies; CSA: clinically suspect arthralgia; RA: rheumatoid arthritis.

a *P*<0.05.

b *P*<0.001.

## Data Availability

The data underlying this article are available from the corresponding author upon reasonable request.

## Statement of clinical significance

It is known that fatigue is a major hurdle for patients with rheumatoid arthritis (RA) and that fatigue has not improved over the past decades. We analyzed fatigue in the phase of arthralgia preceding RA-development: clinically suspect arthralgia (CSA). We showed that fatigue increased towards progression from CSA to RA, indicating the role of inflammation on fatigue in the development of RA. Furthermore, we showed that fatigue is more explained by inflammation in the CSA-phase than in RA. These findings provide clues to further unravel the pathophysiology of fatigue during the trajectory of RA-development, in the light of the multidimensional etiology of this symptom.
