# Supplementary material for: The course of fatigue during the development of rheumatoid arthritis and its relation with inflammation: a longitudinal study
Source: Joint Bone Spine. Author manuscript; Available in PMC 2024 Apr 25. (PMC7615874; doi:10.1016/j.jbspin.2022.105432)
Supplement: S1, S2, S3, S4 [file EMS195435-supplement-S1__S2__S3__S4_.docx]

**Data S1. CSA-cohort**

Patients with CSA were included in the cohort at their first visit at the outpatient clinic, before any blood tests had been performed.[1] In line with national guidelines for general practitioners (GPs), GPs are discouraged to perform ACPA-testing themselves but are encouraged to refer patients in case of any suspicion on imminent RA. Hence inclusion was mostly done without knowledge of the results of additional investigations. At each study visit (0,4,12,24 months), physical joint examination was performed and blood samples were taken for routine laboratory screening, including immunoglobulin M-rheumatoid factor (RF) (positive ≥3.5 IU/mL); and ACPA (anti-CCP2, EliA CCP, Phadia, the Netherlands, positive ≥7 U/mL). In addition, an MRI was made at baseline.

**Data S2. EAC-cohort.**

The Leiden early arthritis clinic (EAC) cohort has previously been described in detail.[2] In short, the EAC-cohort includes consecutive patients with clinical arthritis from at least 1 joint at physical examination by the rheumatologist, and a symptom duration of <2 years. From 2010 onwards MRI was included in the protocol.

**Data S3. Protocol for Magnetic Resonance Imaging (MRI)**

MRI of wrist, metacarpophalangeal (MCP)-, and metatarsophalangeal (MTP)-joints was made with gadolinium contrast enhancement on an MSK-extreme 1.5T extremity MR imaging system (GE, Wisconsin, USA), using a 145mm coil for the foot and a 100mm coil for the hand. Patients were instructed not to use NSAIDs 24 hours prior to MRI. Patient were positioned in a chair beside the scanner, with the hand or foot fixed in the coil with cushions. In the hand (MCP 2-5 and wrist) the following sequence was acquired before contrast administration: T1-weighted fast spin-echo (FSE) sequence in the coronal plane (repetition time (TR) 575ms, echo time (TE) 11.2ms, acquisition matrix 388×288, echo train length (ETL) 2). After intravenous injection of gadolinium contrast (gadoteric acid, Guerbet, Paris, France, standard dose of 0.1mmol/kg) the following sequences were obtained: T1-weighted FSE sequence with frequency selective fat saturation (fatsat) in the coronal plane (TR/TE 700/9.7ms, acquisition matrix 364×224, ETL 2), T1-weighted FSE sequence with frequency selective fat saturation in the axial plane (wrist: TR/TE 540/7.7ms; acquisition matrix 320x192; ETL 2 and MCP-joints: TR/TE 570/7.7ms; acquisition matrix 320x192; ETL 2). The obtained sequences of the forefoot (MTP 1-5 joints) concerned post-contrast images of the foot: T1-weighted FSE fatsat sequence in the axial plane (TR/TE 700/9.5ms; acquisition matrix 364x224, ETL 2) and: T1-weighted FSE fatsat sequence in the coronal plane (perpendicular to the axis of the MTP-joints) (TR/TE 540/7.5ms; acquisition matrix 320x192, ETL 2). Field-of-view was 100mm for the hand and 140mm for the foot. Coronal sequences of the hand had 18 slices with a slice thickness of 2mm and a slice gap of 0.2mm. Coronal sequences of the foot had 20 slices with a slice thickness of 3mm and a slice gap of 0.3mm. All axial sequences had a slice thickness of 3mm and a slice gap of 0.3mm with 20 slices for the wrist, 16 for the MCP-joints and 14 for the foot.

**Data S4. MRI-inflammation; scoring and dichotomization**

Synovitis was scored in line with the Outcome Measures in Rheumatology Clinical Trials (OMERACT) RA MRI scoring (RAMRIS)-method.[3] RAMRIS was not developed to score MTP-joints, however others have previously adapted the RAMRIS to score MTP-joints as well.[4] Tenosynovitis was scored according to the method described by Havaardsholm (also applied at the flexor and extensor tendons at the 2-5 MCP-joints; range 0-3).[5] The synovitis score (range 0-3) was scored based on the volume of enhancing tissue in the synovial compartment (none, mild, moderate, severe) and the tenosynovitis-score (ranged 0-3) was based on the thickness of peritendinous effusion or synovial proliferation with contrast enhancement (normal, <2mm, 2-5mm, >5mm). Presence of inflammation was dichotomized per feature of inflammation (synovitis, tenosynovitis, osteitis) and per location; if the inflammation-score of any feature was higher than present in <5% of age matched healthy controls at the same location, the joint was scored positive for inflammation. This was summed to a total number of joints with inflammation (range 0-10). Baseline MRIs were scored by two experienced readers, blinded to any clinical data. The mean scores of two readers were calculated and in case of disagreement the lower score was used. Inter- and intrareader intraclass correlation coefficients were ≥0.90, as published previously[4].

**Figure S1. Summary scheme of statistical analyses**

**Aim 1) Course of fatigue in CSA**

- 1. CSA-patients developing RA: fatigue in the time prior to arthritis development.


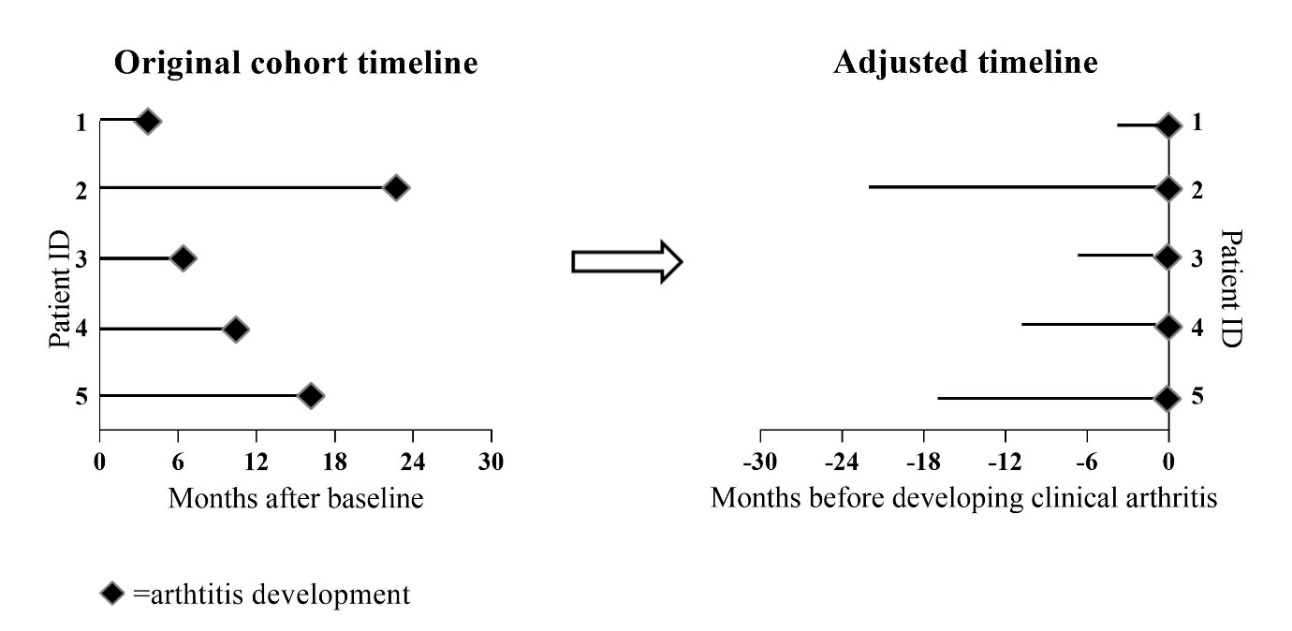
Date of arthritis was transformed into timepoint zero for every patient individually and fatigue-levels prior to their arthritis development were studied. See adjusted timeline below.

- 1. CSA-patients not developing RA: fatigue in the time after baseline visit.

Actual time of follow-up, thus time after baseline visit, was used.

**Aim 2) Association between fatigue and inflammation**

- 1. At CSA-onset, in CSA-patients who developed RA later on.
  2.
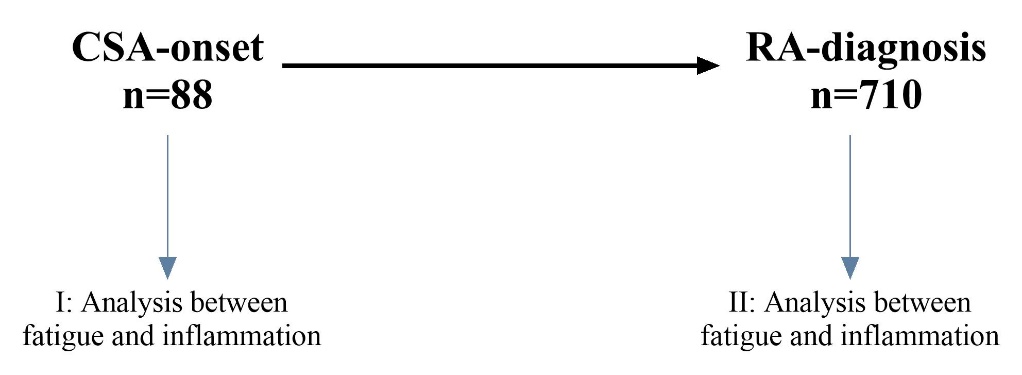
At RA-diagnosis, in RA-patients.

Legend: the group of patients at RA-diagnosis (n=710) is larger than CSA-patients developing RA (n=88), because not all RA-patients have been included through the CSA-cohort.

**Supplemental references**

1. van Steenbergen, H.W., et al., *Characterising arthralgia in the preclinical phase of rheumatoid arthritis using MRI.* Ann Rheum Dis, 2015. **74**(6): p. 1225-32.

2. van Aken, J., et al., *The Leiden Early Arthritis Clinic.* Clin Exp Rheumatol, 2003. **21**(5 Suppl 31): p. S100-5.

3. Østergaard, M., et al., *OMERACT Rheumatoid Arthritis Magnetic Resonance Imaging Studies. Core set of MRI acquisitions, joint pathology definitions, and the OMERACT RA-MRI scoring system.* J Rheumatol, 2003. **30**(6): p. 1385-6.

4. Dakkak, Y.J., et al., *Reliability of Magnetic Resonance Imaging (MRI) Scoring of the Metatarsophalangeal Joints of the Foot according to the Rheumatoid Arthritis MRI Score.* J Rheumatol, 2020. **47**(8): p. 1165-1173.

5. Haavardsholm, E.A., et al., *Introduction of a novel magnetic resonance imaging tenosynovitis score for rheumatoid arthritis: reliability in a multireader longitudinal study.* Ann Rheum Dis, 2007. **66**(9): p. 1216-20.
